# Comparative Analysis of the Base Compositions of the Pre-mRNA 3′ Cleaved-Off Region and the mRNA 3′ Untranslated Region Relative to the Genomic Base Composition in Animals and Plants

**DOI:** 10.1371/journal.pone.0099928

**Published:** 2014-06-18

**Authors:** Xiu-Qing Li

**Affiliations:** Potato Research Centre, Agriculture and Agri-Food Canada, Fredericton, New Brunswick, Canada; Rutgers New Jersey Medical School, United States of America

## Abstract

The precursor messenger RNA (pre-mRNA) three-prime cleaved-off region (3′COR) and the mRNA three-prime untranslated region (3′UTR) play critical roles in regulating gene expression. The differences in base composition between these regions and the corresponding genomes are still largely uncharacterized in animals and plants. In this study, the base compositions of non-redundant 3′CORs and 3′UTRs were compared with the corresponding whole genomes of eleven animals, four dicotyledonous plants, and three monocotyledonous (cereal) plants. Among the four bases (A, C, G, and U for adenine, cytosine, guanine, and uracil, respectively), U (which corresponds to T, for thymine, in DNA) was the most frequent, A the second most frequent, G the third most frequent, and C the least frequent in most of the species in both the 3′COR and 3′UTR regions. In comparison with the whole genomes, in both regions the U content was usually the most overrepresented (particularly in the monocotyledonous plants), and the C content was the most underrepresented. The order obtained for the species groups, when ranked from high to low according to the U contents in the 3′COR and 3′UTR was as follows: dicotyledonous plants, monocotyledonous plants, non-mammal animals, and mammals. In contrast, the genomic T content was highest in dicotyledonous plants, lowest in monocotyledonous plants, and intermediate in animals. These results suggest the following: 1) there is a mechanism operating in both animals and plants which is biased toward U and against C in the 3′COR and 3′UTR; 2) the 3′UTR and 3′COR, as functional units, minimized the difference between dicotyledonous and monocotyledonous plants, while the dicotyledonous and monocotyledonous genomes evolved into two extreme groups in terms of base composition.

## Introduction

After transcription, the three-prime (3′)-most segment of the newly made precursor RNA (pre-RNA) is usually cleaved off [1], [2]. This 3′ cleaved-off region is referred to herein as “3′COR” for the sake of simplicity. The new 3′ end is polyadenylated. There is a 3′ untranslated region (3′UTR) between the coding sequence and the polyadenylation [poly(A)] tail starting position, also often known as the polyadenylation site or poly(A) site. The 3′UTR can include the 3′COR in the broad sense. In practice, however, the 3′UTR is the untranslated 3′ region in the mature messenger RNA (mRNA), because there is no information about the 3′COR in most mRNA sequences. The exact length of the 3′COR at the whole-genome level is unclear; however, some studies have used an approximate length of 200 nucleotide fragments to represent the 3′COR in some yeast genes [3]. Although the function that the pre-mRNA 3′COR performs after transcription termination is unclear, that region is believed to have an important influence on pre-mRNA length and folding as well as on pre-mRNA cleavage. In contrast, the 3′UTR is known to be very gene-specific, to play a critical role in regulating mRNA export, stability, and functionality, and to be critical for the development of living organisms [4]–[6].

Gene-density distribution in fish genomes [7] and human genomes [8] increases with increasing isochore G+C content (GC, C+G or G+C richness). G+C-rich genes are usually more fully expressed than the G+C-poor ones [9]. In vertebrates, introns are poorer in G+C and richer in A+T in comparison with exons [10]. It is widely known that introns are usually less conserved than exons. Within the same genes, however, the G+C content of exons correlates with that of introns [11]. In a previous study, base compositions were analyzed in the 3′UTRs of 271 dicotyledonous and 82 monocotyledonous plant genes [12]; however, these 3′UTRs were only approximate because the poly(A) sites had not been determined. In mammals, there are numerous studies on the motifs around the poly(A) site [13]–[15] as well as some studies on the base composition in the 3′UTR [16] or in the regions both upstream and downstream of poly(A) sites [17]. Little is known about the base-composition differences between the poly(A) site region and the whole genome in different subkingdoms of plants and animals.

In this study, the author analyzed the nucleotide contents of the 201-base region including the 100 bases of the 3′COR and the 100 bases of the 3′UTR immediately adjoining (downstream and upstream, respectively) each poly(A) site in eleven animal species and seven plant species, and compared the results with the nucleotide contents of the corresponding whole genomes (using only complete or nearly complete genomes). The order of these regions are as follows: coding region–3′UTR–Poly(A) tail attachment position–Poly(A) tail starting position [usually called poly(A) site]–3′COR. The mapping used mRNA sequences in the nucleotide database of National Center for Biotechnology Information (NCBI, http://www.ncbi.nlm.nih.gov/) for all the studied species and Illumina RNA-seq reads for four species. The species chosen for this study were the ones with the largest number of unique poly(A) sites mapped on their corresponding genomes. Although the decision to use the 201-base region in this analysis was arbitrary, given that the exact length of the 3′COR is unknown, our intention was to concentrate on the poly(A) site regions and to disregard base-composition effects related to translation termination (upstream part of the 3′UTR) and gene region termination (downstream part of the 3′COR) that can be unrelated to poly(A) site selection.

## Results

### Animal and Plant Genomic Base Compositions

**Figure 1:**
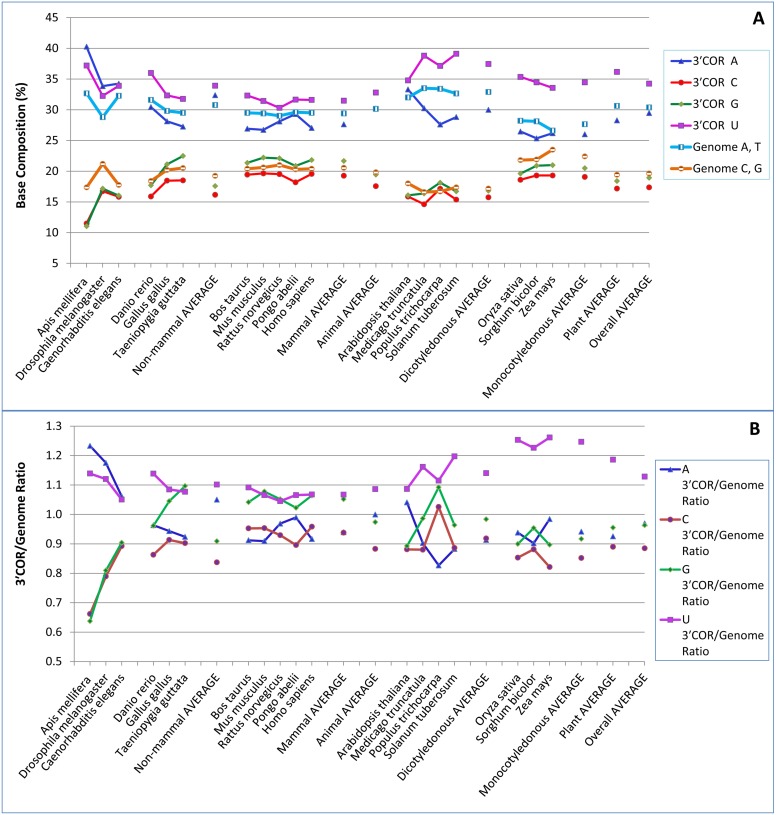
RNA base compositions of the three-prime cleaved-off region (3′COR), represented by the 100 bases downstream of the polyadenylation [poly(A)] site. (A) Base compositions of the 3′COR and the whole genome of each species. (B) 3′COR/genome ratios for base composition. The mapping used NCBI mRNA sequences. The mapped extra copies were eliminated if the sequences in the 100-base three-prime untranslated region were identical. Note that for the 3′COR, U was richest in all species except *Apis mellifera* and *Drosophila melanogaster*, two insect invertebrates, in which A was richest. In *Caenorhabditis elegans* (a nematode), the A, C, G and U contents in the 3′COR are 34.3%, 15.8%, 16.0%, and 33.9%, respectively. Monocotyledonous plants had lower U contents but higher 3′COR/genome ratios in the 3′COR than did dicotyledonous plants.

Both the animal and plant genomes (see Table 1 for the list of species) showed A+T richness in the approximate order A = T>>C = G (Figure 1A). The A and T contents were highest in dicotyledonous plants, lowest in monocotyledonous (cereal) plants, and intermediate in animals (Figure 1A). The C and G contents showed the opposite pattern for the A+T contents, with the highest contents in monocotyledonous plants and the lowest in dicotyledonous plants (Figure 1). Among the eleven animal genomes analyzed in this study, the *Apis mellifera* (honey bees, invertebrates) and *Caenorhabditis elegans* (nematodes, invertebrates) genomes had the highest A and T contents, and the *Drosophila melanogaster* (fruit fly, invertebrates) genome had the lowest.

**Table 1:** List of species compared and the number of mRNA bases in the polyadenylation [poly(A)] site region analyzed.

Species	Common Name	mRNA BasesAnalyzed
Non-mammal animals (invertebrates)		
*Apis mellifera*	Honey bee	37,400
*Drosophila melanogaster*	Fruit fly	190,800
*Caenorhabditis elegans*	Nematode	77,800
Non-mammal animals (vertebrates)		
*Danio rerio*	Zebrafish	1,449,200
*Gallus gallus*	Chicken	157,600
*Taeniopygia guttata*	Zebra finch	161,600
Mammals (vertebrates)		
*Bos taurus*	Cattle	535,800
*Homo sapiens*	Human	6,099,800
*Mus musculus*	Mouse	1,741,800
*Rattus norvegicus*	Rat	2,827,800
*Pongo abelii*	Orangutan	393,000
Dicotyledonous plants		
*Arabidopsis thaliana*	Arabidopsis	886,200
*Medicago truncatula*	A diploid alfalfa	107,200
*Populus trichocarpa*	Poplar	274,200
*Solanum tuberosum*	Potato	27,800
Monocotyledonous plants		
*Oryza sativa*	Rice	138,600
*Sorghum bicolor*	Sorghum	337,000
*Zea mays*	Maize	2,098,000

### Comparison between pre-mRNA 3′-Cleaved-off Regions (3′COR) and Genomes

All of the animal and plant species had a 3′COR base-composition pattern of U>>A>>G>C, except for the two insect invertebrates (honey bee and fruit fly), which had the pattern A>U>>C = G, according to the poly(A) sites determined by the mapping of NCBI mRNA sequences to their reference genomes (Figure 1A). In *C. elegans* 3′COR, A and U contents were found to be very similar (Figure 1A). On average, the U content in the 3′COR was highest in dicotyledonous plants, lowest in mammal animals, and intermediate in monocotyledonous plants (Table 2; Figure 1A). The vertebrate non-mammal animals had higher U contents and lower C contents in the 3′COR than did the mammals (Table 2; Figure 1). The C content was higher in mammals and monocotyledonous plants, and lower in dicotyledonous plants and non-mammal animals (Figure 1A). The A content was generally much higher than the G and C contents in all species (Figure 1A), but lower (except in the two invertebrates) than the U content (Figure 1A). Monocotyledonous plants had the lowest A contents overall for all species groups. There was no general difference between animals and dicotyledonous plants in A content (Figure 1A). The G contents were similar to, but generally higher than, the C contents in all species, except for the two invertebrates and *Arabidopsis thaliana*, in which the C and G contents were approximately the same (Figure 1A).

**Table 2:** ANOVA-Duncan’s multiple range tests of base U contents of different subkingdoms.

Region and subkingdom	No. of species	Mean of uracil (U)contents (%)	Duncan test^a^
3′UTR_Dicots	4	39.88	A
3′COR_Dicots	4	37.45	B
3′UTR_Monocots	3	35.34	BC
3′UTR_Non-mammals	6	34.93	C
3′COR_Monocots	3	34.47	C
3′COR_Non-mammals	6	33.92	CD
Genome_Dicots	4	32.84	CDE
3′UTR_Mammals	5	31.87	DEF
3′COR_Mammals	5	31.46	DEF
Genome_Non-mammals	6	30.78	EF
Genome_Mammals	5	29.48	FG
Genome_Monocots	3	27.63	G

a :Means with the same letter are not significantly different (P<0.05).

In comparison with the genome, the 3′COR showed consistently higher U contents in all species, slightly lower A contents, and generally lower C contents (Figure 1A). The G contents in the 3′COR were appreciably higher than the genomic G contents in mammals and substantially lower than the genomic G contents in the monocotyledonous plants (Figure 1A). ANOVA and Duncan multiple-range test confirmed that monocot plants had the lowest genomic U content among all subkingdoms, but monocot 3′COR had a relatively high average U content (Table 2).

The 3′COR/genome ratio for U content (i.e., the ratio obtained by dividing the U content in the 3′COR by the whole-genome T content) was significantly highest among the 3′COR/genome ratios in monocotyledonous plants, intermediate in dicotyledonous plants, and lowest in animals (Figure 1B; Table 3). In most species, the 3′COR/genome ratio for C content was the smallest ratio for the four bases (Figure 1B). The 3′COR/genome ratios for A and G contents showed considerable variation. However, in monocotyledonous plants, only U was consistently overrepresented to a considerable extent; the other three bases (A, C, and G) were underrepresented in the 3′COR of monocotyledonous plants (Figure 1B).

**Table 3:** ANOVA and Duncan’s multiple range tests of the region/genome ratios of U contents.

Region and subkingdom	No. ofspecies	Mean of 3′UTR/genome or 3′COR/genome ratios of U contents	Duncan test^a^
3′UTR_Monocots	3	1.30	A
3′COR_Monocots	3	1.25	AB
3′UTR_Dicots	4	1.20	B
3′COR_Dicots	4	1.14	C
3′UTR_Non-mammal animals	6	1.13	C
3′UTR_Mammals	5	1.10	CD
3′COR_Non-mammal animals	6	1.10	CD
3′COR_Mammals	5	1.07	D

a :Means with the same letter are not significantly different (P<0.05).

**Figure 2:**
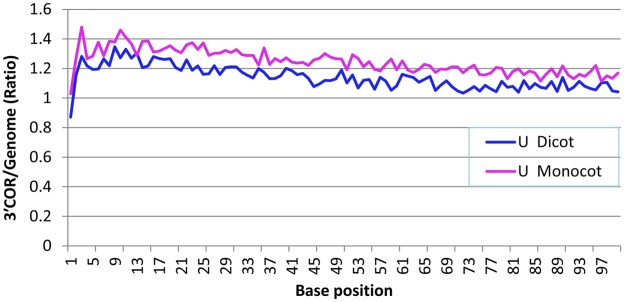
RNA base composition of each position in the 100-nucleotide region of the three-prime cleaved-off region (3′COR) downstream of the polyadenylation [poly(A)] site. The mapping used NCBI mRNA sequences. Note that both dicotyledonous and monocotyledonous plants had similar variation tendencies, particularly for the first few bases. In comparison with dicotyledonous plants, however, monocotyledonous plants always had a greater 3′COR/genome ratio for U frequency at each position among all the mapped unique poly(A) sites.

At each position along the 100 bases of the 3′COR, the 3′COR/genome ratio for U content was consistently higher than 1.0 in both monocotyledonous and dicotyledonous plants, except for the first base after the poly(A) site in dicotyledonous plants (Figure 2). This means that U was more frequent at every position in the 100-base region in the 3′COR than in the whole genome (Figure 2). However, the 3′COR/genome ratio was consistently higher in monocotyledonous plants than in dicotyledonous plants (Figure 2). Although the first two bases had the smallest ratios, the 3′COR/genome ratio was higher in regions closer to the poly(A) site than in the regions farther downstream of the poly(A) site (Figure 2).

### Comparison between mRNA 3′-Untranslated Regions (3′UTR) and Genomes

**Figure 3:**
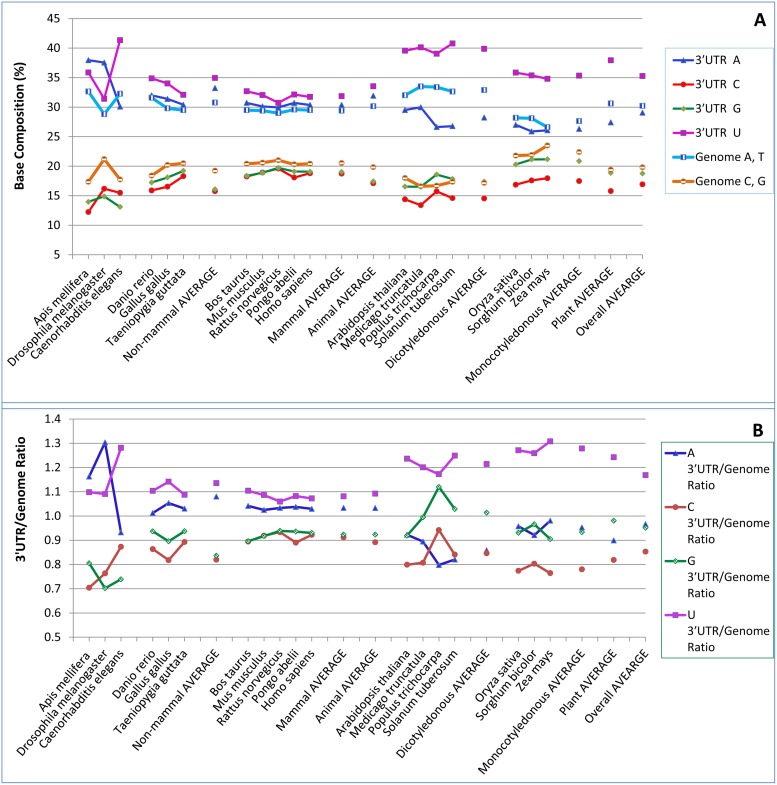
RNA base compositions of the three-prime untranslated region (3′UTR), represented by the 100 bases upstream of the polyadenylation [poly(A)] site. (A) Base compositions of the 3′UTR and the whole genome of each species. (B) 3′UTR/genome ratios of base composition. The mapping used NCBI mRNA sequences. The mapped extra copies were eliminated if the sequences in the 100-base 3′UTR were identical. Note that for the 3′UTR, compared with other nucleotides, U was the richest in all species except *Apis mellifera* and *Drosophila melanogaster*, two insect invertebrates, in which A was the richest (Figure 3A). Monocotyledonous plants had lower U contents but higher 3′UTR/genome ratios in the 3′UTR than did dicotyledonous plants. The U content in the 3′UTR was significantly different between dicotyledonous plants and monocotyledonous plants and between non-mammal animals and mammals according to one-way ANOVA and Duncan’s multiple range test at the *P*<0.05 level (Table 2). The C content in the 3′UTR was significantly different between non-mammal animals (15.82%) and mammals (18.73%) and between dicotyledonous plants (14.53%) and monocotyledonous plants (17.47%) according to the same tests.

Like the 3′CORs, the mRNA 3′UTRs showed a base-composition pattern of U>>A>>G>C in all species except the two insect invertebrates (honey bees and fruit flies) (Figure 3A). The U content in the 3′UTR was highest in dicotyledonous plants, lowest in animals, and intermediate in monocotyledonous plants (Figure 3A; Table 2). In addition, like the 3′CORs, the 3′UTRs had higher U and lower C contents in the non-mammal animals than in the mammals (Figure 3A). In the 3′UTRs, the C content was highest in mammals, lowest in dicotyledonous plants, and intermediate in monocotyledonous plants and non-mammal animals (Figure 3A). The A content was generally lower than the U content but much higher than the G and C contents, except in the two insects, which had the highest A contents (Figure 1A). The A content was lowest in monocotyledonous plants, highest in animals, and intermediate in dicotyledonous plants (Figure 3A). The G contents were similar to, but usually higher than, the C contents (Figure 1A).

In comparison with the genome, the 3′UTR had the highest U content in all species (except for the fruit fly and the honey bee, two invertebrates), a slightly higher A content in animals, and an appreciably lower A content in plants (Figure 3A). The G content in the 3′UTR was lower than the genomic G content in animals and monocotyledonous plants but approximately similar in dicotyledonous plants, which had the lowest genomic C and G contents among the subkingdoms covered in this study (Figure 3A). The U content in 3′UTR was consistently higher on average than in 3′COR in every plant and animal subkingdom, even though the difference was not significant in mammals (Table 2 and Table S1).

The 3′UTR/genome ratio for U content was highest in monocotyledonous plants, intermediate in dicotyledonous plants, and lowest in animals (Figure 3B; Table 3). The 3′UTR/genome ratio for A content was greater than 1.0 in animals, but clearly lower than 1.0 in plants (Figure 3B). In monocotyledonous plants in the 3′UTR, only U was strongly overrepresented; the other three bases (A, C, and G) were underrepresented (Figure 3B). In the analysis of each position along the 3′UTR, the area within approximately 30 bases of the poly(A) site was found to be highly variable in base composition, but U was always the most dominant of the four bases in the region farther upstream (data not shown). Strong overrepresentation of U content was obvious at most positions in the 3′UTR. The base U was more overrepresented in 3′UTR than in 3′COR in terms of the region/genome ratio in every subkingdom (Table 3 and Table S2).

### Comparison of Dicotyledonous and Monocotyledonous Plants about Poly(A) Sites Mapped with NCBI mRNA

The dicotyledonous plants show the highest U contents in the 3′COR and 3′UTR, possibly because they have the highest genomic U contents and moderate overrepresentation of U (Figures 1A and 3A). In monocotyledonous plants, however, the high U contents in these two regions are attributable mainly to the strongest overrepresentation of U (Table 3), given that they had the lowest genomic U contents (Table 2; Figure 3B). Even though overrepresentation of U was strongest in monocotyledonous plants, the actual U contents in the 3′COR and 3′UTR were lower than in dicotyledonous plants (Table 2). The base-composition differences between dicotyledonous and monocotyledonous plants in the 3′COR were 4.0%, −3.3%, −3.7%, and 3.0% for A, C, G, and U, respectively (in terms of contents in dicots minus the contents in monocots), which were smaller than the corresponding differences in the whole genome (5.3%, −5.2%, −5.2%, and 5.2%, for A, C, G, and T, respectively) (Figure 1A). The base-composition differences between dicotyledonous and monocotyledonous plants in the 3′UTR were 4.0%, −3.3%, −3.7%, and 3.0% for A, C, G, and U, respectively, which were also smaller than the differences in the whole genome (Figure 3A).

### Base Composition of the mRNA 3′UTR Region 50 Bases Away from Poly(A) Sites

It is known that the region within 25 bases from the poly(A) site usually has specific A- or U-rich motifs [18]. These motifs may affect the calculated A- or U- contents of the 100 base 3′UTR. To verify whether the 3′UTR is still U-rich (in most species) or A-rich (in the two insect species) without this motif-rich regions, the author also analyzed the base composition of the 50-base UTR region that was 50 bases upstream away from the poly(A) site. Similar base composition orders between A, C, G, and U were confirmed between this 50-base region and the 100-base region of the 3′UTR in all the species: U was richest for all the species except for the two insects (honey bee and fruit fly) (Table 4). The U/A ratio in this 50-base region was found to be significantly higher in plants than in animals (Table 4).

**Table 4:** Base compositions of the mRNA three-prime untranslated region (3′UTR), represented by the 50-base region that is 50 bases away from the polyadenylation [poly(A)] site^a^.

Species	A (%)	C (%)	G (%)	U (%)	U/A ratio^b^
Non-mammal animals (invertebrates)					
*Apis mellifera*	36.57	13.24	14.93	35.26	0.96
*Drosophila melanogaster*	34.13	17.55	16.45	31.87	0.93
*Caenorhabditis elegans*	26.75	18.15	13.84	41.26	1.54
Non-mammal animals (vertebrates)					
*Danio rerio*	28.92	16.91	18.72	35.46	1.23
*Gallus gallus*	27.99	17.83	19.88	34.3	1.23
*Taeniopygia guttata*	27.17	19.71	21.48	31.64	1.16
Mammals (vertebrates)					
*Bos taurus*	27.15	20.17	20.59	32.1	1.18
*Homo sapiens*	26.95	20.41	21.45	31.19	1.16
*Mus musculus*	26.34	20.96	21.24	31.46	1.19
*Rattus norvegicus*	26.56	21.03	22.1	30.31	1.14
*Pongo abelii*	27.87	18.92	21.46	31.75	1.14
Dicotyledonous plants					
*Arabidopsis thaliana*	27.95	14.59	17.4	40.06	1.43
*Medicago truncatula*	29.02	13.53	17.59	39.86	1.37
*Populus trichocarpa*	25.41	16.11	19.73	38.75	1.53
*Solanum tuberosum*	25.47	15.18	18.56	40.79	1.60
Monocotyledonous plants					
*Oryza sativa*	25.42	17.77	21.58	35.24	1.39
*Sorghum bicolor*	24.3	18.22	22.73	34.75	1.43
*Zea mays*	24.77	18.52	22.47	34.23	1.38

a :Poly(A) sites were mapped using NCBI mRNA sequences.

b :The mean U/A ratio is significantly higher in plants than in animals according to Student’s *t*-test (P = 0.0006, with a two tailed distribution and two-sample equal variance model).

### Illumina Reads-mapped 3′UTR and 3′COR

**Figure 4:**
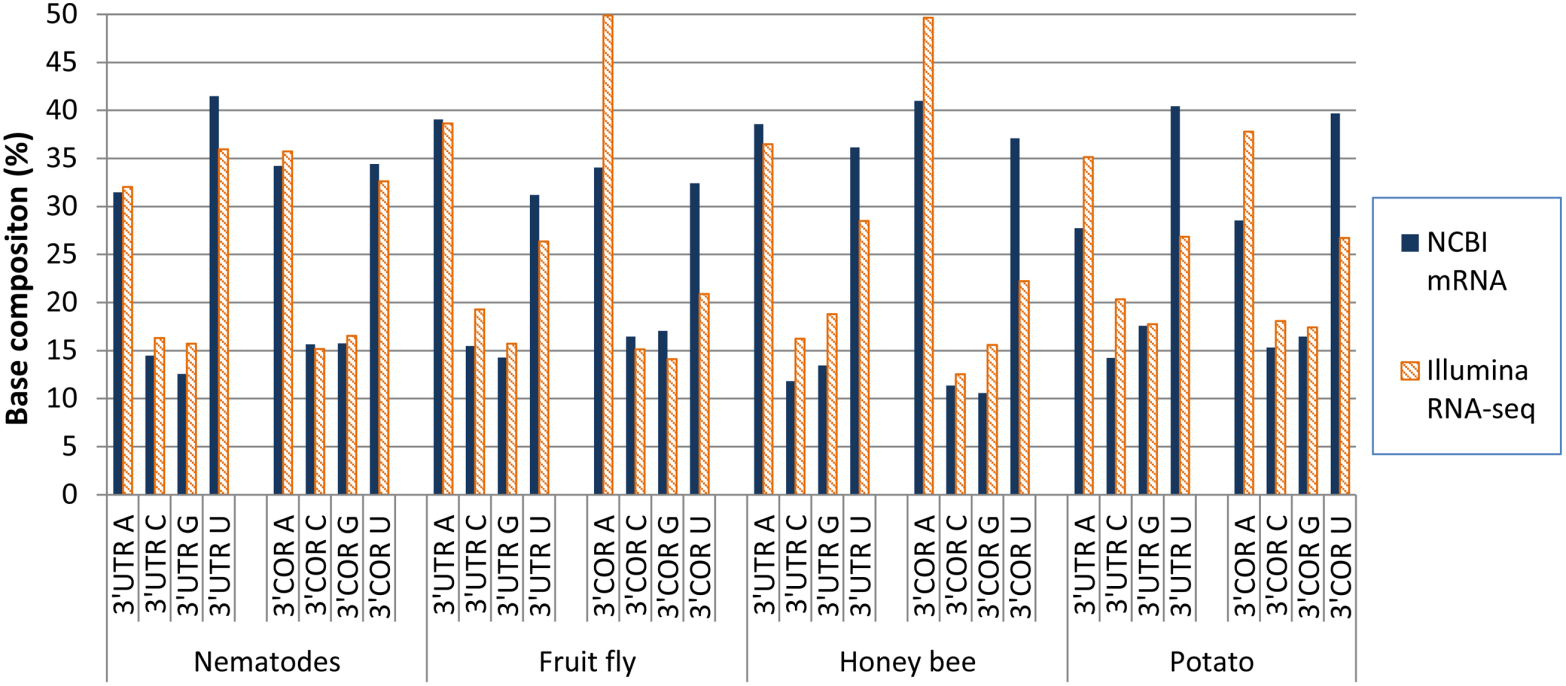
Base composition around poly(A) sites in comparison between NCBI mRNA-based mapping and Illumina HiSeq reads-based mapping. Nematode: *Caenorhabditis elegans*. Fruit fly: *Drosophila melanogaster*. Honey bee: *Apis mellifera*. Potato: *Solanum tuberosum* (Group Phureja, diploid).

**Figure 5:**
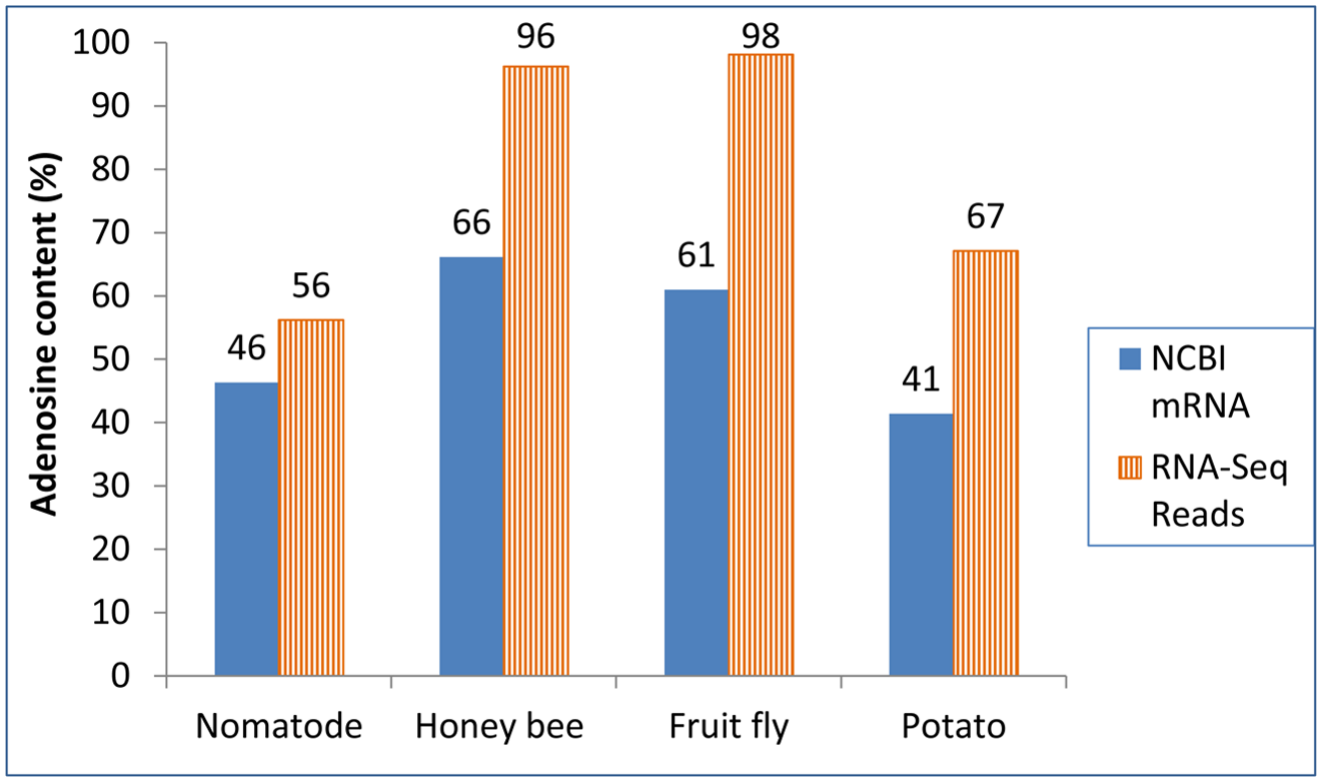
Adenosine content (i.e., A content) for the first six bases of 3′COR. This 6 bases include the poly(A) site and the 5 immediately downstream bases. The A content represents the average percentage of A in the 6-base region of all mapped poly(A) sites. The RNA-Seq reads were from TruSeq using Illumina HiSeq 2000 or 2500. Information about the Sequence Read Achives (SRA) transcriptomic files can be found in Table 5. NCBI mRNA and RNA-Seq reads were significantly different in this 6-base region according the Excel “ChiTest” in each of these four species (P<0.0001). Note that this six-base region showed higher adenosine content in mapping with RNA-Seq reads than mapping with NCBI mRNA.

Illumina deep sequencing data of mRNA (RNA-Seq) were analyzed for poly(A) sites in nematode (*C. elegans*), fruit fly, honey bee, and potato (Table 5 and Figure 4). Compared with the NCBI mRNA-based analysis, the base composition of the 3′COR region from Illumina RNA-seq reads showed the following (Figure 4): 1) much higher overrepresentation of A in all the species; 2) lower U contents; 3) less different between C, G, and U. In the 3′COR, the 6 bases counted from the poly(A) site were more predominantly A in mapping with Illumina TruSeq RNA-Seq reads than mapping with NCBI mRNA sequences (Figure 5). The extremely high average A content of the 6 base positions in the poly(A) site regions strongly suggests that the Illumina Tru-Seq reads-based mapping was sensitive to internal priming. In the 3′UTR region, the U-predominance estimated by Illumina RNA-Seq is generally lower than that estimated by NCBI-mRNA based mapping (Figure 4).

**Table 5:** Description of Illumina RNA HiSeq files.

Species	Common name	SRAnumber^a^	Illumina seqsystem	No. ofBases	Mappedunique poly(A) sites
*Caenorhabditis elegans*	Nematode	SRR1174011	HiSeq 2500	4.4 G	579
*Drosophila melanogaster*	Fruit fly	SRR988055	HiSeq 2500	2.1 G	547
*Apis mellifera*	Honey bee	SRR789759	HiSeq 2500	17.8 G	7871
*Solanum tuberosum*	Potato	SRR1232054	HiSeq 2000	9.8 G	1290

a :Nematiode, fruit fly, and honey bee RNA-seq files were downloaded from NCBI Sequence Read Archives (SRA). The potato RNA-Seq file was from the present study. Three additional nematode RNA-Seq files (SRR1174012, SRR1174013, and SRR1174014) and an additional fruit fly RNA-Seq file (SRR988056) were also analyzed, and similar results were obtained as from the presented files (data not shown).

## Discussion

Mapping using Illumina RNA-Seq reads was found to be very likely more sensitive to internal priming than mapping using non-deep sequencing-generated mRNA sequences as shown in Figures 4 and 5. This is likely because, at the cDNA library construction stage, Illumina uses random hexamers in the step of the first strand cDNA synthesis and very low annealing temperatures: 25°C for the first strand and 16°C for the second strand synthesis (Illumina Cat #RS-930-1001). Whereas, NCBI mRNA sequences were mainly from traditional cDNA libraries such as Strategene cDNA Library in which it uses usually 18 mer oligo dT and an annealing temperature of 42°C (See the manuals of Catalog 200400, ZAP-cDNA Synthesis Kit; Cat. 200401 for cDNA Synthesis Kit; and Cat. 200450 for ZAP-cDNA Gigapack III Gold Cloning Kit) (www.stratagene.com). Another issue of using reads from some deep sequencing nano-technologies such as Illumina is that mRNA/cDNA are fragmented to small pieces before adding adapters. Some internal multiple-A stretches can have chance to show as poly(A) tail at the 3-prime end of sequence reads. This is an obvious issue in addition to internal priming. The generally higher A content in both 3′COR and 3′UTR in Illumina read mapping is consistent with the expectation that A-rich genomic regions have more multiple-A stretches than non-A-rich regions. Traditional sequencing technologies for mRNA or expressed sequence tag (EST) sequencing do not fragment cDNA and therefore can avoid this fragmentation-created poly(A) tails. Further research is required to eliminate the internal priming and cDNA fragmentation issues before RNA-seq reads can be used in poly(A) site mapping with a reliability close to that of the NCBI mRNA sequences. The mRNA sequences were more suitable than Illumina RNA-Seq reads in poly(A) site mapping for species-level evaluation of 3′COR and 3′UTR base composition in this study (Figure 4). The following discussion, therefore, mainly on poly(A) sites mapped using NCBI mRNA sequences.

With respect to other poly(A) site studies in animals and plants, the design of the present investigation is novel owing to the combined use of the following five aspects: (1) complete or nearly complete genomes; (2) a large number of species; (3) RNA sequences from the NCBI Nucleotide database instead of single-pass-based sequences from databases of expressed sequence tags (ESTs) or sequence reads; (4) mRNA–genome alignment with zero tolerance for mismatches; and (5) comparison of base compositions between poly(A) site regions and the whole genome.

Most of the previous studies on this topic were based on the accumulated databases of DNA clone sequences [12], [16], [19], because at the time not many complete or nearly complete genome sequences of animals and plants were available. Our approach largely avoids the problem of over-contribution from redundant genomic DNA clone sequences in the NCBI database. The quality of the transcript sequences we used is generally more reliable than that of single-pass reads, because most mRNA sequences submitted to the NCBI mRNA database are supposed to have been verified by sequencing from both directions, particularly if a poly(A) tail is included in the submitted sequences. The NCBI’s mRNA databases are typically smaller than the databases of ESTs and other single-run reads; however, the higher quality of the mRNA can largely compensate for the use of a smaller database, provided only species with sufficient numbers of mapped poly(A) sites for statistical tests are compared and provided the comparisons made are mainly between groups of species. Furthermore, we applied a zero tolerance approach to mismatches in our transcript–genome alignment, which is much stricter than the mismatch tolerance of 10% applied in a previous study on the topic [16]. This zero-tolerance approach to mismatches can help to prevent or minimize ambiguity in mapping. As well, the mapping done in the present study was based on the 100-nucleotide upstream sequence, which is much more stringent than the 60-nucleotide sequence mapping approach used in the previous study [16]. Compared with our previous characterization of the poly(A) site starting position and the poly(A) site attachment position [20], in this study we removed any redundant poly(A) sites after mapping to minimize the inflation effects of unexpressed alleles. These stricter mapping criteria reduced the number of poly(A) sites and the number of species to be compared but greatly increased the reliability of the mapping and minimized the dilution effects from fault sites.

Likely because complete genome sequences were unavailable, previous base-composition studies focused mainly on the 3′UTR and did not analyze the 3′COR in multiple animals and plants [12], [16]. Although the genomes that recently became available have greatly contributed to the characterization of both the upstream and downstream regions around poly(A) sites in mammals [17], little information is available to use in comparing the base composition of the 3′UTR and 3′COR with that of the whole genome. In the current study, we examined the base composition of the 3′COR and 3′UTR relative to the genomic base composition in mammals as well as non-mammal animals, dicotyledonous plants, and monocotyledonous plants.

To reach conclusions on general differences between animals and plants and between dicotyledonous and monocotyledonous plants, it is critically important to include a sufficiently large number of species in the analysis. Although the study on EST and DNA clones in a previous study [16] generated valuable information about poly(A) sites, no conclusions could be reached about differences between animals and plants and between dicotyledonous and monocotyledonous plants, because only one dicotyledonous species (*Arabidopsis thaliana*), one monocotyledonous species (rice), and three animal species (fruit fly, mouse, and human) were studied. In our study, we used the RNA–genome alignment approach and analyzed both the 3′UTR and 3′COR in eleven animal species and seven plant species.

Although we examined more species in each subkingdom than did previous studies, the numbers are still not large enough to permit comparisons within sub-groups such as insects and non-insects or woody and herbaceous plants. For example, honey bee (*Apis mellifera*) and fruit fly (*D. melanogaster*) were the only two insects in the invertebrates studied, and *Populus trichocarpa* was the only tree species. This is because we used only complete or nearly complete genomes. In future, research can be undertaken to verify whether other tree and insect species have base compositions in the 3′UTR and 3′COR similar to those of the two species we studied, once more insects and trees have been completely sequenced. The larger variation among species in the “non-mammal animals” group than in the “mammals” group (Figures 1 and 3) is likely attributable to the fact that the “non-mammal animals” group is very diverse and included both invertebrate and vertebrate animals.

Interestingly, we found that insect invertebrates (honey bees and fruit flies) preferred A over U in 3′UTR and 3′COR (Figures 1 and 3), which is the opposite of what we found for all non-insect animals. Among the 17 plant and animal species analyzed, these two insects invertebrates clearly stood out from the other 15 species in terms of the A/U ratio. The species-specificity in the A/U ratio may suggest genetic influence on the base composition in the 3′UTR and 3′COR. Since the two insect invertebrates gave similar results (opposite to those for vertebrates and nematodes), the results are unlikely to represent an artifact associated with the relatively smaller number of poly(A) sites mapped on the honey bee genome. Although further research may lead to more optimal settings and thus improve RNA-seq sequence read analysis, the NCBI-mRNA-based approach still has its rightful place because of its higher sequence quality and its usually broader coverage of tissues and treatments relative to the currently available sequence reads. In the present study, the high base-composition similarity of the poly(A) site region among species within subkingdoms and the general difference between subkingdoms suggest that the results are unlikely due to coincidence or bias and that the data must reflect the true biology of these subkingoms/species.

One of the important features of this study is the comparison between the 3′COR and 3′UTR and the whole genome. Most of the previous studies on this topic described the nucleotide contents of the 3′UTR without examining differences or similarities between that region and the corresponding genomes. It is unclear whether the base-composition difference in the 3′UTR between animals and plants is a simple reflection of differences in their whole-genome base compositions. Although the G content in the 3′UTR was found to be very similar in *Arabidopsis thaliana* and fruit flies [16], we found that the G content in the 3′UTR and the genomic G content were similar in *A. thaliana* but the G content in the 3′UTR was much lower than the genomic G content in fruit flies. This suggests that there is a bias against G in the 3′UTR in fruit lies (Figure 3A). This kind of difference can only be identified through a comparative study of the 3′UTR and the genome.

The most consistent feature of the base compositions of both the 3′COR and 3′UTR in both animals and plants was found to be lower C contents in these regions than in the whole genomes (Figures 1A and 3A). The C contents were the lowest among all the four types of nucleotides in the 3′COR and 3′UTR regions but differed significantly between subkingdoms. These results appear to be somewhat similar to the AT richness found in the introns and intergenic sequences of two animals in a previous study [21]. In the present study, however, the GC poorness in the 3′COR and 3′UTR is caused mainly by C poorness, because the G content varies depending on the species; in fact, G was usually much higher than C in the 3′UTR in both monocotyledonous and dicotyledonous plants (Figures 1 and 3).

Interestingly, each animal and plant subkingdom showed distinct characteristics in terms of base composition (Figures 1A and 3A). Different groups of living organisms have their own sets of unique genes. For example, immune system genes play high-ranked and conserved roles in mammals but are not conserved in nematodes [22]. As well, each subkingdom may differ in its own transposons and its regulation of DNA mutation/repair systems. Further research is required to investigate in what way unique genes, transposons, and DNA mutation/repair systems contribute to base-composition differences. It is known that cellular selection favouring translation differs between G+C-rich and G+C-poor classes of genes [23]. Given that base-composition patterns are known to differ between animals and plants in the 3′COR and 3′UTR (as shown in this study), it is logical to expect that different selection mechanisms apply to animals and plants. Further research is needed to find out what these selections are in living organisms. Since U (usually the most dominant base) was consistently overrepresented and C (usually the least dominant) was consistently underrepresented in the 3′COR and 3′UTR, regardless of their respective content variation in the whole genome, U and C must play important roles in both poly(A) region selection and interaction with the poly(A) complex.

It is likely that G+C richness affects gene length in vertebrate G+C-rich isochores [24]. We found differences in C or G contents between animal and plant genomes. It is unclear whether these C or G differences influence the lengths of the 3′COR and 3′UTR. Built on the present study of the 201 nucleotides in this untranslated–cleaved-off region (100 bases in the 3′COR), future research could analyze the base composition of more-distant downstream regions relative to the whole genome. This will make it possible to determine the approximate difference in 3′COR length between animals and plants.

Plant chromosomes are more often characterized by the predominance of genes on the same-direction than are animal chromosomes [25]. It would be interesting to see whether gene direction has any relationship with the base composition in the 3′COR and 3′UTR. Base composition and genome or chromosome size are correlated in various organisms [26], and genome and chromosome sizes are known to strongly impact gene direction on chromosomes during the increase of life complexity [25]. The region of 3′COR and 3′UTR are either a part of, or close to, the gene end. This gene region creates a certain level of repeats between genes, in terms of conserved base composition patterns. Further research is required to investigate whether the base composition in this 3′COR-3′UTR gene end region affects DNA recombination and consequently impact the gene direction rearrangement on chromosomes.

Plants have extreme patterns of genomic base composition in comparison with animals. Dicotyledonous plants were found to have extremely high genomic A and T contents and extremely low C and G contents (Figure 1A). In contrast, monocotyledonous plants were found to have the lowest genomic A and T contents and the highest genomic C and G contents among the four subkingdoms (non-mammal animals, mammals, dicotyledonous plants, and monocotyledonous plants) (Figure 1A). Interestingly, among all the plant and animal species analyzed, monocotyledonous plants had the lowest T contents in genome (27.63%, Table 2; Figure 1A) but the highest increase in the U contents in terms of mRNA 3′UTR/genome ratio (1.30; Table 3; Figure 1B) and mRNA 3′COR/genome ratio (1.25; Table 3; Figure 3B). Whereas dicotyledonous plants had a higher genomic T contents on average than monocot plants (Figure 3A) but significant smaller increases in the U contents in the same mRNA regions (3′UTR/genome ratio = 1.20 and 3′COR/genome ratio = 1.14; Table 3; Figures 1B and 3B). These adjustments made the U content difference between monocot and dicot plants smaller in the 3′UTR and 3′COR region than in the genome. Our hypothesis is that the important function of the 3′UTR and 3′COR makes it less likely that these regions will undergo mutation during evolution (even though they are very rich in A+T) than most other regions of the genome. Further research is needed to determine whether this means that the T content is too low in monocotyledonous genomes and must be enriched to a certain degree to permit the 3′COR and 3′UTR to function properly in monocotyledonous cells.

Among the species within subkingdoms, the base compositions in the 3′COR (Figure 1A) were more similar than the 3′COR/genome ratios (Figure 1B). This pattern of similarity in 3′CORs among species and of weaker similarity in their 3′COR/genome ratios was particularly obvious among mammals and among monocotyledonous plants (Figure 1). The base compositions in the 3′UTR (Figure 3A) were also more similar than the 3′UTR/genome ratios (Figure 3B). These results appear to suggest that the 3′COR and the 3′UTR are evolutionarily more stable than the genomes in terms of base composition changes. Further research is required to gain a better understanding of the similarities and differences among species between the 3′COR and 3′UTR regions and the genomes. This evolutionary trend drove the analyzed sequences in the same direction: the most frequent nucleotide was U, followed by A, G, and C, in most of the animals and plants studied. Although the U content was slightly lower than the A content in the 3′COR and 3′UTR in the two insect invertebrates (honey bee and fruit fly), U was consistently much more frequent than G and C in these regions in all species. The results also suggest that the nucleotide content in these 3′COR and 3′UTR sequences evolved nearly independently of the rest of the genome. The knowledge acquired about the base compositions of the whole genome and the 3′COR and 3′UTR in eleven animals and seven plants may stimulate further research aimed at interpreting the results.

The analysis used highly reliable data, characterized the base-composition of the 3′COR and 3′UTR in comparison with that of the whole genome, and identified clear differences between dicotyledonous and monocotyledonous plants and between non-mammal animals and mammals.

## Materials and Methods

### Genomes and mRNA Sequences

Nucleotide sequences of complete genomes were downloaded in FASTA format from the NCBI website at http://www.ncbi.nlm.nih.gov/sites/genome. Most of the animal and plant species for which both complete genomes (serve as the reference genomes) and large mRNA databases were available in NCBI were screened as described previously [20], and the species that had sufficient poly(A)-tailed mRNA in NCBI were used for detailed analysis.

### Mapping mRNA on Genomes

The screening of poly(A) tailed mRNA and the mapping of poly(A) tailed mRNA to corresponding genomes were carried out essentially as described in the previous study of the dinucleotide covering the pre-mRNA 3′end cleavage site [20]. Only the transcripts that each had a poly(A) tail of at least 12 continuous A’s at the 3′ end were used in this analysis. The 100 bases immediately upstream of the poly(A) site were used to screen the mRNA datasets to eliminate any redundant copies. Each sequence in the final dataset of poly(A)-tailed mRNA was unique. These sequences were mapped to the reference genomes of their corresponding species with zero tolerance for mismatches.

Several species for which sequenced genomes were available were not included in the final comparative study, because a) the number of mapped unique poly(A) sites was too small for comparison, b) the mRNA dataset of the species (i.e., *Macaca mulatta* and *Pan troglodytes*) had a large number of computation predicted mRNA or c) many of their mapped poly(A) sites had 12 or more A’s and were potentially more susceptible to internal priming (i.e., *Macaca mulatta*, *Pan troglodytes*, and *Sus scrofa*) than were most other species.

Most species used in comparison in this study had likely very low percentage of internal priming, partly due to the use of high quality sequences from the mRNA database and partly due to the very strict settings used in this mapping. For example, only 0.3% percentage of mapped mRNAs in plants had 12 A’s [20]. Its potential modification of the content (percentage) of each specific nucleotide (A, C, G, and U) in the whole mRNA pool would be less than 0.1% on average. Whereas, the actually U content difference detected between 3′UTR and whole genome in monocot plants was about 8% (Table 2). The internal priming issue had no power to change the conclusions in this mRNA study. The internal priming issue has been described and discussed in detail previously [20].

The post-mapping treatment that was applied differed from that in our previous study [20] as follows: To minimize overrepresentation from duplicated gene copies, the extra copies were eliminated if the 100-base sequences (i.e., 3′UTR) upstream of the poly(A) sites were identical. Thus, every mRNA sequence and every poly(A) site analyzed were unique in terms of this 100-nucleotide 3′UTR. All of the poly(A) tail screening, mRNA–genome alignment, and base-composition counting were assisted by Perl scripts.

The computation included the following steps: a) searched for poly(A) tailed mRNA using the requirements described in the Methods section; b) eliminated the duplicated mRNA sequences using the 100 bases upstream of the poly(A) site; aligned the poly(A)-tailed mRNA sequences with the reference genome of the same species with zero tolerance for mismatches; c) kept only one site from identical multiple sites based on the 100-base upstream nucleotide sequence; d) eliminated the species that had high proportions of predicted mRNA in the mapped sequences; e) eliminated the species that had multiple-A stretches immediately after poly(A) sites on the genome; f) counted the base composition for each position of the 201 bases for each mapped mRNA; g) counted the average base composition of each region [upstream and downstream of the poly(A) site] for the pooled mapped-sequences; and h) compared them with the whole genome base composition.

### Nucleotide Contents

The A, C, G, or T nucleotide content in each genome is the percentage of A, C, G, or T in the total nucleotide number of the genome accumulated from all the chromosomes. In the case of species for which the complete chromosome sequences or pseudomolecules were unavailable, we used large scaffolds. The pre-mRNA 3′COR nucleotide contents were from the 100-base genomic sequence downstream of, but not including, the poly(A) tail starting position [i.e., the genomic or pre-mRNA nucleotide corresponding to the first A of the poly(A) tail]. The 3′UTR base compositions were from the 100 mRNA bases upstream of, but not including, the poly(A) tail starting position.

### DNA Preparation, Illumina Sequencing, and Sequence Read Analysis

Plants of a doubled monoploid potato line DM1-3 516R44 (*S. tuberosum* Group Phureja) [27] were growing in the greenhouse. Total RNA was prepared from roots using RNeasy Plant Mini Kit (Qiagen Cat. 74903). A TruSeq mRNA cDNA library was constructed using this RNA and then sequenced by pair-end 100 cycles using Illumina HiSeq 2000 at the Genome Quebec-McGill University Innovation Centre. Reads were processed by removing the adapters, poor quality regions and too short ones using Trimmomatic [28] with setting of MINLEN:50 and TRAILING:30. Detail of transcriptomic analysis will be published elsewhere. Sequence reads with 12 A’s at the 3′ end were used in alignment to the potato (Group Phureja) reference genome (PGSC_DM_v4.03 downloaded from http://solanaceae.plantbiology.msu.edu/pgsc_download.shtml).

Illumina RNA-Seq files of nematiode, fruit fly, and honey bee were downloaded from NCBI Sequence Read Archives (SRA) (http://www.ncbi.nlm.nih.gov/sra/). SRA file IDs are listed in Table 5. Sequence reads with 12 A’s at the 3′ end were considered polyadenylated.

The Illumina RNA-seq read mapping was based on the 80-base region immediately upstream of the poly(A) site. The author eliminated the duplicated RNA-seq reads for the 80-base sequences upstream of the poly(A) site before starting mapping. The mapping was with zero tolerance of mismatch. The redundant copies of the mapped poly(A) sites in terms of the sequence of the same 80 bases were also eliminated after mapping.

### Statistical Analysis

The content of each type of base (A, C, G, and U), the 3′UTR/genome ratios, and the 3′COR/genome ratios were compared among non-mammal animals, mammals, dicotyledonous plants, and monocotyledonous plants at the *P*<0.05 level by one-way ANOVA and Duncan’s multiple range test at the *P*<0.05 level using SAS Enterprise Guide, version 4.3. The U/A ratio mean comparison between plants and animals was according to Student’s *t*-test (in Excel 2010) with a two tailed distribution and two-sample equal variance model. ChiTest (in Excel 2010) was used to test the A content differences for the 6-bases in the poly(A) site region between mapped NCBI mRNA and mapped RNA-Seq reads.

## Supporting Information

Table S1 **ANOVA-Duncan’s multiple range tests of base U contents of different subkingdoms (classifying animals into invertebrates and vertebrates).**
(DOCX)

Table S2 **ANOVA and Duncan’s multiple range tests of the region/genome ratios of U contents (classifying animals into invertebrates and vertebrates).**
(DOCX)

## References

[1] Chambers A, Old R (1988) RNA 3′ cleavage and polyadenylation in oocytes and unfertilized eggs of *Xenopus laevis* . Dev Biol 125: 237–245. 2892746 10.1016/0012-1606(88)90207-2

[2] Yamanaka S, Yamashita A, Harigaya Y, Iwata R, Yamamoto M (2010) Importance of polyadenylation in the selective elimination of meiotic mRNAs in growing *S. pombe* cells. EMBO J 29: 2173–2181. 20512112 10.1038/emboj.2010.108 PMC2905246

[3] Birse CE, Minvielle-Sebastia L, Lee BA, Keller W, Proudfoot NJ (1998) Coupling termination of transcription to messenger RNA maturation in yeast. Science 280: 298–301. 9535662 10.1126/science.280.5361.298

[4] Juge F, Zaessinger S, Temme C, Wahle E, Simonelig M (2002) Control of poly(A) polymerase level is essential to cytoplasmic polyadenylation and early development in *Drosophila* . EMBO J 21: 6603–6613. 12456666 10.1093/emboj/cdf633 PMC136937

[5] Schisa JA, Strickland S (1998) Cytoplasmic polyadenylation of *Toll* mRNA is required for dorsal-ventral patterning in Drosophila embryogenesis. Development 125: 2995–3003. 9655821 10.1242/dev.125.15.2995

[6] Barkoff A, Ballantyne S, Wickens M (1998) Meiotic maturation in *Xenopus* requires polyadenylation of multiple mRNAs. EMBO J 17: 3168–3175. 9606198 10.1093/emboj/17.11.3168 PMC1170655

[7] Costantini M, Auletta F, Bernardi G (2007) Isochore patterns and gene distributions in fish genomes. Genomics 90: 364–371. 17590311 10.1016/j.ygeno.2007.05.006

[8] Rayko E, Jabbari K, Bernardi G (2006) The evolution of introns in human duplicated genes. Gene 365: 41–47. 16356663 10.1016/j.gene.2005.09.038

[9] Akashi H (2001) Gene expression and molecular evolution. Curr Opin Genet Dev 11: 660–666. 11682310 10.1016/s0959-437x(00)00250-1

[10] Fortes GG, Bouza C, Martínez P, Sánchez L (2007) Diversity in isochore structure among cold-blooded vertebrates based on GC content of coding and non-coding sequences. Genetica 129: 281–289. 16897446 10.1007/s10709-006-0009-2

[11] Bulmer M (1987) A statistical analysis of nucleotide sequences of introns and exons in human genes. Mol Biol Evol 4: 395–405. 3447014 10.1093/oxfordjournals.molbev.a040453

[12] Kochetov AV, Syrnik OA, Rogozin IB, Glazko GV, Komarova ML, et al (2002) Context organization of mRNA 5′-untranslated regions of higher plants. Mol Biol 36: 510–516. 12173469

[13] Beaudoing E, Freier S, Wyatt JR, Claverie JM, Gautheret D (2000) Patterns of variant polyadenylation signal usage in human genes. Genome Res 10: 1001–1010. 10899149 10.1101/gr.10.7.1001 PMC310884

[14] Hu J, Lutz CS, Wilusz J, Tian B (2005) Bioinformatic identification of candidate *cis*-regulatory elements involved in human mRNA polyadenylation. RNA 11: 1485–1493. 16131587 10.1261/rna.2107305 PMC1370832

[15] Borodulina OR, Kramerov DA (2008) Transcripts synthesized by RNA polymerase III can be polyadenylated in an AAUAAA-dependent manner. RNA 14: 1865–1873. 18658125 10.1261/rna.1006608 PMC2525947

[16] Graber JH, Cantor CR, Mohr SC, Smith TF (1999) *In silico* detection of control signals: mRNA 3′-end-processing sequences in diverse species. Proc Natl Acad Sci USA 96: 14055–14060. 10570197 10.1073/pnas.96.24.14055 PMC24189

[17] Derti A, Garrett-Engele P, MacIsaac KD, Stevens RC, Sriram S, et al (2012) A quantitative atlas of polyadenylation in five mammals. Genome Res 22: 1173–1183. 22454233 10.1101/gr.132563.111 PMC3371698

[18] Li X-Q, Du D (2014) Motifs types, motif locations, base composition patterns, and structure around the RNA polyadenylation site in microorganisms, plants, and animals. BMC Evol Biol: (Accepted with revision). 10.1186/s12862-014-0162-7 PMC4360255 25052519

[19] Tian B, Graber JH (2012) Signals for pre-mRNA cleavage and polyadenylation. Wiley Interdiscip Rev RNA 3: 385–396. 22012871 10.1002/wrna.116 PMC4451228

[20] Li X-Q, Du D (2013) RNA polyadenylation sites on the genomes of microorganisms, animals, and plants. PLoS ONE 8: e79511. 24260238 10.1371/journal.pone.0079511 PMC3832601

[21] Jabbari K, Bernardi G (2004) Comparative genomics of *Anopheles gambiae* and *Drosophila melanogaster* . Gene 333: 183–186. 15177694 10.1016/j.gene.2004.02.038

[22] Du D, Lee CF, Li X-Q (2012) Systematic differences in signal emitting and receiving revealed by PageRank analysis of a human protein interactome. PLoS ONE 7: e44872. 23028653 10.1371/journal.pone.0044872 PMC3446998

[23] Mukhopadhyay P, Basak S, Ghosh TC (2007) Nature of selective constraints on synonymous codon usage of rice differs in GC-poor and GC-rich genes. Gene 400: 71–81. 17629420 10.1016/j.gene.2007.05.027

[24] Duret L, Mouchiroud D, Gautier C (1995) Statistical analysis of vertebrate sequences reveals that long genes are scarce in GC-rich isochores. J Mol Evol 40: 308–317. 7723057 10.1007/BF00163235

[25] Li XQ, Du D (2012) Gene direction in living organisms. Sci Rep 2: 982.

[26] Li X-Q, Du D (2014) Variation, evolution, and correlation analysis of C+G content and genome or chromosome size in different kingdoms and phyla. PLoS ONE 9: e88339. 24551092 10.1371/journal.pone.0088339 PMC3923770

[27] Xu X, Pan S, Cheng S, Zhang B, Mu D, et al (2011) Genome sequence and analysis of the tuber crop potato. Nature 475: 189–195. 21743474 10.1038/nature10158

[28] Lohse M, Bolger AM, Nagel A, Fernie AR, Lunn JE, et al (2012) RobiNA: A user-friendly, integrated software solution for RNA-Seq-based transcriptomics. Nucleic Acids Res 40: W622–W627. 22684630 10.1093/nar/gks540 PMC3394330

