# Supplementary material for: Comparative Analysis of the Base Compositions of the Pre-mRNA 3′ Cleaved-Off Region and the mRNA 3′ Untranslated Region Relative to the Genomic Base Composition in Animals and Plants
Source: PLoS One. 2014 Jun 18;9(6):e99928. doi: 10.1371/journal.pone.0099928 (PMC4062462; doi:10.1371/journal.pone.0099928)
Supplement: Table S1 — ANOVA-Duncan’s multiple range tests of base U contents of different subkingdoms (classifying animals into invertebrates and vertebrates). (DOCX) [file pone.0099928.s001.docx]

**Table S1.** ANOVA-Duncan’s multiple range tests of base U contents of different subkingdoms (classifying animals into invertebrates and vertebrates).

| Region and subkingdom | No. of species | Mean of uracil (U) contents (%) | Duncan test*^a^* |
| --- | --- | --- | --- |
| 3′UTR_Dicots | 4 | 39.88 | A |
| 3′COR_Dicots | 4 | 37.45 | AB |
| 3′UTR_Invertebrates | 3 | 36.20 | CB |
| 3′UTR_Monocots | 3 | 35.34 | CBD |
| 3′COR_Monocots | 3 | 34.47 | CDE |
| 3′COR_Invertebrates | 3 | 34.46 | CDE |
| Genome_Dicots | 4 | 32.84 | FDE |
| 3′UTR_vertebrate | 8 | 32.54 | FE |
| 3′COR_vertebrate | 8 | 32.18 | FEG |
| Genome_Invertebrates | 3 | 31.25 | FG |
| Genome_vertebrate | 8 | 29.79 | HG |
| Genome_Monocots | 3 | 27.63 | H |

*^a^*:Means with the same letter are not significantly different (P < 0.05).
