# Supplementary material for: Comparative Analysis of the Base Compositions of the Pre-mRNA 3′ Cleaved-Off Region and the mRNA 3′ Untranslated Region Relative to the Genomic Base Composition in Animals and Plants
Source: PLoS One. 2014 Jun 18;9(6):e99928. doi: 10.1371/journal.pone.0099928 (PMC4062462; doi:10.1371/journal.pone.0099928)
Supplement: Table S2 — ANOVA and Duncan’s multiple range tests of the region/genome ratios of U contents (classifying animals into invertebrates and vertebrates). (DOCX) [file pone.0099928.s002.docx]

**Table S2.** ANOVA and Duncan’s multiple range tests of the region/genome ratios of U contents (classifying animals into invertebrates and vertebrates).

| Region and subkingdom | No. of species | Mean of 3′UTR/genome or 3′COR/genome ratios | Duncan test*^a^* |
| --- | --- | --- | --- |
| 3′UTR_Monocots | 3 | 1.30 | A |
| 3′COR_Monocots | 3 | 1.25 | AB |
| 3′UTR_Dicots | 4 | 1.20 | CB |
| 3′UTR_Invertebrates | 3 | 1.16 | CD |
| 3′COR_Dicots | 4 | 1.14 | ED |
| 3′UTR_vertebrate | 8 | 1.10 | EF |
| 3′COR_Invertebrates | 3 | 1.09 | EF |
| 3′COR_vertebrate | 8 | 1.08 | F |

*^a^*:Means with the same letter are not significantly different (P < 0.05).
